# An engineered yeast cytosine deaminase with improved catalytic activity and stability for macrophage-mediated enzyme/prodrug therapy

**DOI:** 10.1038/s42003-025-08931-x

**Published:** 2025-11-13

**Authors:** Jiale Zheng, Jiahao Zhou, Kristen Wing Yu Yung, Qipeng Hu, Marianne M. Lee, Michael K. Chan

**Affiliations:** School of Life Sciences and Center of Novel Biomaterials, The Chinese University of Hong Kong, Shatin, Hong Kong SAR China

**Keywords:** Biomaterials - proteins, Immobilized enzymes, Drug delivery

## Abstract

Utilization of yeast cytosine deaminase (yCD) to activate the prodrug 5-fluorocytosine (5-FC) to 5-fluorouracil (5-FU) at the target site is an attractive strategy for overcoming the narrow therapeutic index of 5-FU. Nevertheless, protein delivery of yCD is challenging in part due to its thermal instability. Herein, we have rationally engineered a mutant yCD by replacing Met100 situated at the active site entry with the bulkier histidine to hinder potential oxidation of the active site Cys91. The engineered yCD-Met100His exhibits significantly enhanced activity and thermal stability. yCD-M100H is then genetically fused to the crystal-forming protein Cry3Aa to generate Cry3Aa-yCD-M100H fusion crystals to facilitate the enzyme’s uptake into macrophages. The resulting Cry3Aa-yCD-M100H-loaded macrophages exhibit excellent penetration into tumor spheroids and readily convert 5-FC to 5-FU leading to efficacious cancer cell killing. This study showcases a promising route for stabilizing yCD and the feasibility of enzyme-internalized macrophages to serve as tumor-specific enzyme/prodrug activators.

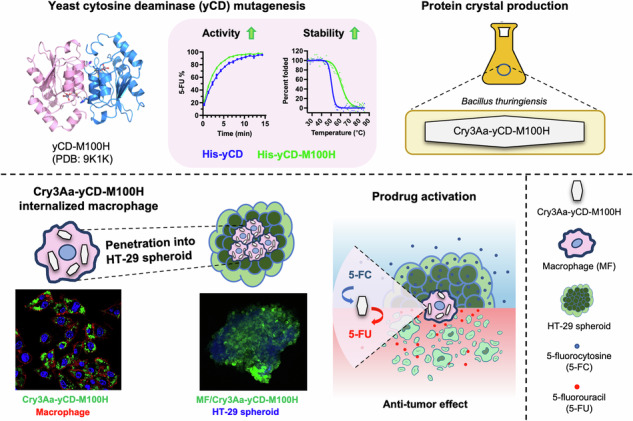

## Abstract (web summary)

An engineered yCD with enhanced oxidation resistance and intracellular stability encapsulated in macrophages showed excellent spheroid penetration and cancer cell killing, demonstrating its use as tumor-specific enzyme/prodrug activator.

## Introduction

5-fluorouracil (5-FU) is a potent chemotherapeutic used widely in clinic for the treatment of various cancers. As with most chemotherapeutics, its indiscriminate toxicity to both cancerous and normal tissues leads to severe side effects in patients, and greatly limits the administration of the planned dose level at which optimal clinical response could be achieved^1^. 5-fluorocytosine (5-FC), an antifungal agent used clinically to treat fungal infections, has a much safer toxicity profile, and can be deaminated into 5-FU by cytosine deaminase.

Cytosine deaminases (CD) catalyze the hydrolytic deamination reaction of cytosine to uracil, and are found only in prokaryotes and fungi, but not mammalian cells. Extensive research has since been conducted to develop CD/5-FC as an enzyme/prodrug system for cancer therapy^2–5^. Compared with bacterial CDs, which exhibit a significant reduction in substrate affinity and catalytic efficiency, the yeast CD from *Saccharomyces cerevisiae* (yCD) has a much lower *K*_m_ for 5-FC and a higher conversion efficiency. However, yCD is thermolabile^6,7^ and loses its activity rapidly at physiological temperatures. This undesirable property greatly hinders its therapeutic utility. Thus, different strategies, including the introduction of stabilization mutations^8–10^, immobilization^11^, and conjugation to antibodies^12^ have been explored to enhance the enzyme’s thermostability and lifetime.

Structural studies of yCD have shown that its active site contains a tightly bound zinc ion critical for catalytic activity, and is coordinated by His62 and two cysteine residues Cys91 and Cys94, and a hydroxyl group bridged to a second tetrahedrally coordinated zinc bound by three water molecules^13^. A close examination of the yCD structure reveals a small cavity near the active site entrance of each subunit, which we hypothesized might facilitate the access of surrounding bulk solvent to the active site cysteines, leading to their oxidation. We therefore devised a rational design strategy to improve yCD’s stability by incorporating a mutation that could shield Cys91 and thereby hinder its potential oxidation.

Limited tumor infiltration is a major hurdle that must be overcome to achieve meaningful therapeutic efficacy. Macrophages have been exploited for their intrinsic tumor-targeting and deep penetration abilities to deliver therapeutics into tumors, including the hypoxic core regions that are difficult to reach^14^. Although promising outcomes have been reported by many studies^14^, multiple challenges remain for using macrophages as cell-mediated drug carriers, particularly for delivery of protein-based cargoes. These include the non-toxicity of the cargo protein and its limited perturbation on the macrophage’s functional properties; the physiochemical properties of the cargo, including its size, shape, and surface properties to ensure efficient drug loading; and the release of the drug from the carrier in order to act on the cancer cells^14–17^.

The stability of the cargo protein within the macrophage is another major factor. As a case in point, cell-mediated delivery of the gene of yCD as a prodrug activator for 5-FC has been widely reported^18–20^, but the delivery of yCD protein via cellular carriers is rare—perhaps due in part to the thermolability of yCD making it particularly challenging to preserve the encapsulated enzyme long enough to reach and act on its target. Our group has previously reported the development of a protein delivery platform based on Cry3Aa protein that naturally forms crystal in the bacterium *Bacillus thuringiensis*^21,22^. One advantageous feature of this platform is its excellent ability in protecting the cargo protein from proteolysis and acidic pH degradation. In addition, the Cry3Aa and Cry3Aa fusion crystals have shown to be readily taken up by macrophages^21^. Thus, we surmised that the Cry3Aa crystalline framework would be well-suited for the protection of yCD from the degradative elements inside macrophages.

In this study, we describe the rational engineering of a yCD-M100H mutant and its development as a prodrug/enzyme agent. Using X-ray crystallographic and biochemical studies, we validate our hypothesis and design strategy for the protection of yCD’s active site cysteine towards enhancing its lifetime and preservation of catalytic activity. Through the use of Cry3Aa crystal platform to which the improved yCD-M100H is genetically fused, we demonstrate the resultant fusion crystals attained enhanced intracellular stability that allows for their encapsulation in macrophages with no adverse effect to the yCD’s activity and macrophage’s tumor-targeting and penetration properties. The anti-tumor effect of the macrophage-encapsulated Cry3Aa-yCD-M100H observed only in the presence of 5-FC, and their deep penetration into the tumor spheroids establish the potential of these macrophage-encapsulated Cry3Aa-yCD-M100H as a promising prodrug/enzyme anti-cancer system that can minimize non-specific chemotoxicity while effectively kill cancer cells.

## Results and discussion

### Engineering a thermostable and active yeast cytosine deaminase

Previous studies have reported that yeast cytosine deaminase (yCD) has a much shorter half-life at physiological temperature than bacterial CDs due in part to it being more thermolabile^6,23^. We thus sought to engineer a more stable yCD while preserving its high catalytic activity. An analysis of the *Saccharomyces cerevisiae* yCD structure^13^ revealed a cavity formed near the active site in each subunit that would allow facile O_2_ transport from the bulk solution to the active sites, and which we hypothesized could react with oxygen, leading to the facile enzyme inactivation observed. A close examination of the structure identified a methionine residue, M100, located at the entry of this cavity, which we surmised could be replaced with a bulkier residue to provide steric hindrance to block any incoming O_2._ Computational modeling suggested that leucine and histidine could provide the desired shielding, and thus the corresponding mutants, yCD-M100H and yCD-M100L, were generated for evaluation. In addition, previous studies had reported a yCD triple mutant that exhibited a 30-fold half-life enhancement at 50 °C^9^. Thus, we also combined the aforementioned leucine or histidine mutation with the triple mutations of the reported triple mutant (yCD-TM) to generate the mutants, yCD-TMH and yCD-TML, for the screening experiments (Supplementary Fig. 1).

**Fig. 1:**
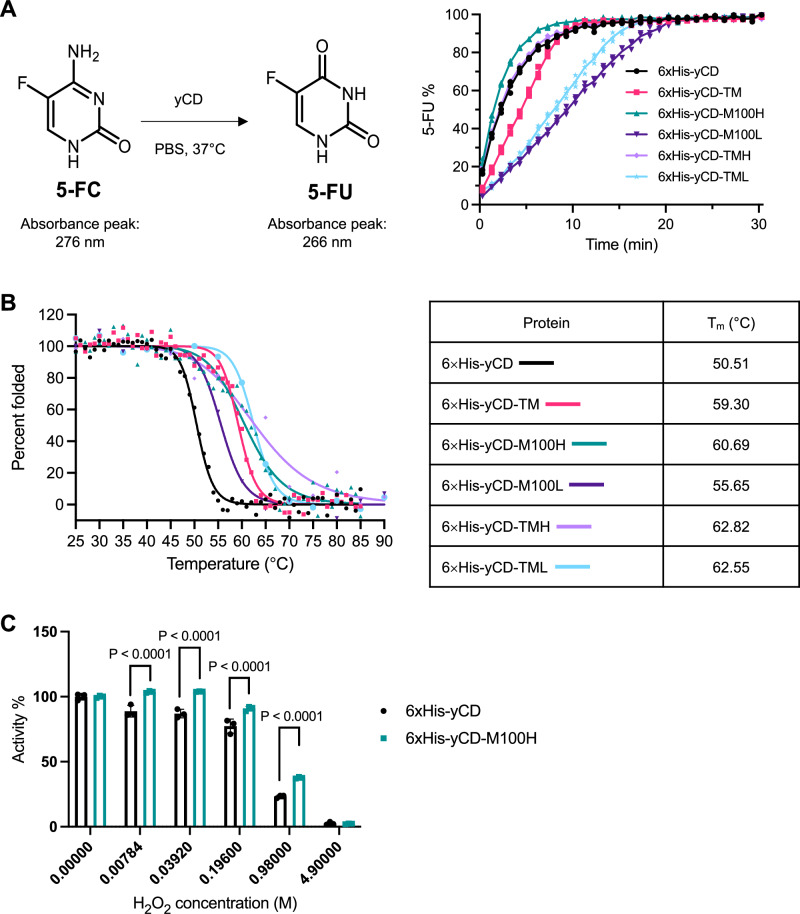
Effect of methionine substitution of yeast cytosine deaminase. **A** Enzymatic activities of the purified his-tagged yCD and its mutant proteins at 37 °C in PBS. 250 nM of the protein was incubated with 1 mM 5-FC substrate, and the conversion of 5-fluorocytosine (5-FC) to 5-fluorouracil (5-FU) was monitored by measuring the absorbance at 276 nm and 266 nm, which was used to derive the % of 5-FU produced (*n* = 3). **B** Thermal stability of the purified wild-type yCD and its mutant proteins assessed by circular dichroism spectroscopy at different temperatures. The readings were recorded at 220 nm from 25 °C to 85 °C and the measurements were used in determining the percentage of folded protein. The melting temperature (T_m_) was determined using GraphPad Prism software. **C** The oxidation resistance abilities of wild-type yCD and mutant yCD-M100H were assessed by pre-incubating 500 nM enzymes in PBS with hydrogen peroxide (H_2_O_2_) at concentrations ranging from 7.84 mM to 4.90 M for 10 min. After the addition of catalase to remove residual H_2_O_2_, the residual enzymatic activities were determined using 1 mM 5-FC normalized to the corresponding enzymes without hydrogen peroxide treatment (*n* = 3). Data were shown as mean ± SD.

All constructs were successfully expressed and purified (Supplementary Fig. 2A). Enzymatic activities converting 5-FC to 5-FU at 37 °C indicated that the single mutant yCD-M100H was the most efficient, as it could complete the conversion in half of the time required by the wild-type yCD and the triple mutant yCD-TM (Fig. 1A). Furthermore, this amino acid substitution of methionine with histidine was able to enhance the yeast CD’s thermostability by 10 °C (*T*_m_ = 60.69 °C), which was comparable to that achieved by the triple mutant yCD-TM (*T*_m_ = 59.30 °C) (Fig. 1B), whose mutations have also been reported to improve yCD’s thermostability^9^. All other yCD variants tested showed enhanced thermostability as indicated by their high *T*_m_ (Fig. 1B), but at the cost of their activity (Fig. 1A).

Given the significant enhanced enzymatic activity and thermostability exhibited by yCD-M100H, the effect of the substitution of Met100 with the bulkier histidine, which we hypothesized would provide better shielding to the active site Cys91, was investigated for its ability to hinder oxidative assault. Towards this end, yCD-M100H and wild-type yCD were exposed to increasing concentrations of hydrogen peroxide (H_2_O_2_), and their ability to convert 5-FC to 5-FU was evaluated. Compared with yCD, which exhibited a drop in activity even at the lowest H_2_O_2_ concentration tested (7.84 mM), yCD-M100H was able to maintain 100% activity for up to a concentration of H_2_O_2_ of 39.2 mM, and was significantly more active across all concentrations tested (Fig. 1C). To obtain structural confirmation for this observation, we proceeded to determine the structure of yCD-M100H protein by X-ray crystallography.

### Structural studies of the yeast cytosine deaminase mutant yCD-M100H

**Fig. 2:**
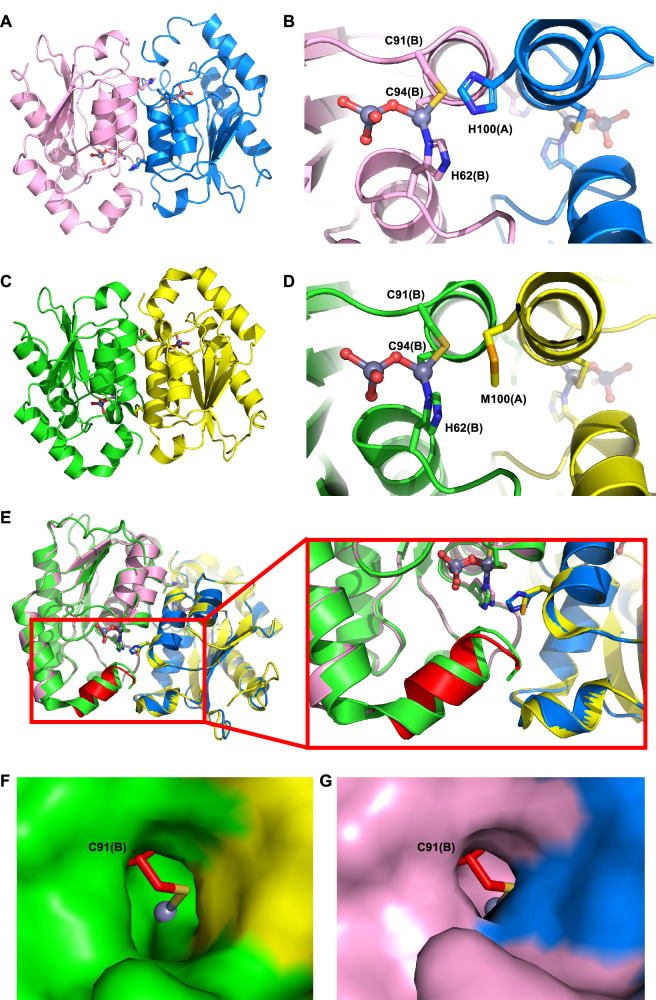
Structural comparison of the mutant M100H and wild-type yeast cytosine deaminase. **A** Overall structure of mutant yeast cytosine deaminase (yCD-M100H) dimer with the A-subunit colored in blue and the B-subunit in pink. Residues 7-158 are fitted in the model. **B** Structure of a yCD-M100H active site consisting two Zn^2+^ ions, one of which is coordinated by the active site residues, His62, Cys91, and Cys94, as well as a presumed hydroxide which bridges to the second zinc whose remaining tetrahedral coordination is filled by water molecules. The M100 residue is also shown to provide its relative location to the active site Cys91. **C** Structure of the wild-type yeast cytosine deaminase dimer with the A-subunit colored in yellow and the B-subunit in green. **D** Active site of wild-type yeast cytosine deaminase. **E** Overlay of yCD-M100H and wild-type yCD (PDB ID: 1OX7) structures with the subunits colored as in panels A and C except for the C-terminal helix of yCD-M100H of subunit B, which is colored in red. The two enzymes share similar conformations except for the C-terminal helices, which are shifted away from the active site in yCD-M100H. View of the protein surface covering the active site residue Cys91 for **F** wild-type yCD and **G** its M100H mutant. The M100H substitution results in greater shielding of Cys91 from reaction than the original methionine residue, as evidenced by the reduced solvent-accessible surface area of Cys91.

The 1.44 Å structure of yCD-M100H (Fig. 2A, B and Table 1) revealed that the protein exists as a mono-dimer that is structurally similar to the original yCD (PDB: 1OX7) (Fig. 2C, D), with an overall RMSD of 0.415 Å based on pairwise alignment by the General Efficient Structural Alignment of Macromolecular Targets (GESAMT) from the CCP4 suite^24^ (Supplementary Fig. 3). The major deviation exists at the C-terminus where the C-terminal helix is being pushed outward in the yCD-M100H structure (Fig. 2E), which is also the segment exhibiting the highest RMSD as analyzed by GESAMT (Supplementary Fig. 3). The histidine mutation does not affect the active site conformation nor the interaction of the catalytic Zn^2+^ ion with the active site ligands (Fig. 2A, B). In concurrence with our hypothesis, the histidine substitution indeed provides more coverage for the active site residue Cys91 along with the Zn^2+^ ion. The more rigid and bulkier side chain of histidine significantly reduces the solvent-accessible surface exposure area of Cys91, thereby protecting it from getting oxidized (Fig. 2F, G). This observation and the resultant effect on yCD’s activity and stability (Fig. 1) are in line with emerging evidence showing that oxidation of catalytic cysteines can have a significant impact on the structural and functional properties of proteins^25,26^.

**Table 1:** Data collection and refinement statistics

	yCD-M100H
Data collection	
Space group	P4_3_
Cell dimensions	
*a*, *b*, *c* (Å)	79.08, 79.08, 55.91
*α*, *β*, *γ* (°)	90, 90, 90
Resolution (Å)	50 (1.44)^a^
*R*_merge_	0.087 (0.36)
*I*/σ*I*	31.3 (7.7)
Completeness (%)	99.8 (98.4)
Redundancy	11.4 (8.9)
Refinement	
Resolution (Å)	1.44
No. reflections	62628
*R*_work_/*R*_free_	12.77 / 14.42
No. atoms	5755
Protein	2397
Ligand/ion	4
Water	331
*B*-factors	
Protein	29.84
Ligand/ion	28.37
Water	29.45
R.m.s. deviations	
Bond lengths (Å)	0.35
Bond angles (°)	0.63

^a^Data from one crystal. Values in parentheses are for highest-resolution shell.

Additionally, it has been reported that the C-terminal helix is important in facilitating yCD’s conformational dynamics crucial for catalysis^27,28^. Previous crystal structures^13^ as well as ours show that the C-terminal tail forms an α-helical lid directly over the entrance of the active site cavity (Fig. 2E), acting as a gate in controlling substrate entry and product release. The latter has been found to be the rate-limiting step^9,28^. Yao and colleagues showed that by extending the C-terminal helix with an additional 68-residue helix derived from myosin resulted in a yCD variant whose C-terminal helix was slightly swung out from the active site and which exhibited much higher catalytic efficiency. The authors attributed this enhanced efficiency to increased C-terminal helix dynamics^29^.

In light of these findings, the kinetic behavior of the wild-type yCD and the mutant yCD-M100H was investigated to gain further insight into the impact of the histidine substitution on enzyme activity. Mutant yCD-M100H was significantly more active than its wild-type counterpart, exhibiting a much lower K_m_ and higher *k*_*cat*_ values than the wild-type’s (Table 2). Taken together, it appears that the mutation to histidine aids in enhancing the conformation dynamics of the C-terminal helix, which presumably enables more efficient exit of the product from the active site, while providing concomitant protection to the active site Cys91 from being oxidized.

**Table 2:** Kinetic parameters of wild-type and mutant yeast cytosine deaminase

	K_*m*_ (mM)	V_*ma*x_ (mM prod s^−1^)	*k*_*cat*_ (s^−1^)
Wild-type yCD	6.68	0.0005433	2.2
yCD-M100H	1.68	0.0031447	12.6

### Incorporation of the engineered yeast cytosine deaminase into Cry3Aa protein crystal platform

**Fig. 3:**
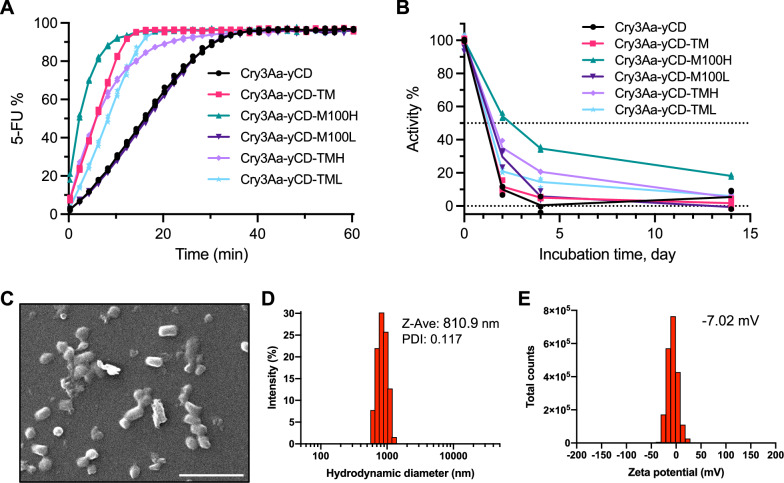
Characterization of Cry3Aa-yCD crystals. **A** Enzymatic activities of the various Cry3Aa fusion crystals were determined by monitoring the conversion of 5-FC to 5-FU. 500 nM of the fusion crystals were reacted with 1 mM of 5-FC in PBS at 37 °C, and the absorbance at 276 to 266 nm was measured at different times (*n* = 3). **B** Kinetic stability of Cry3Aa-yCD fusion crystals and its variants were determined by pre-incubating the crystals in PBS at 37 °C for different lengths of time prior to measuring their residual activity. The residual activity was normalized to the corresponding protein crystals without pre-incubation (*n* = 3). **C** Scanning electron micrographs of Cry3Aa-yCD-M100H crystals. Magnification: 10,000x. Scale bar: 5 μm. **D** Size distribution of the purified Cry3Aa-yCD-M100H protein crystals as measured by dynamic light scattering. **E** Histogram of the zeta potential of Cry3Aa-yCD-M100H crystals measured on a Malvern Zetasizer Nano ZS90.

Proteolysis is a major factor affecting the serum stability of protein therapeutics. We have previously demonstrated that the Cry3Aa crystal framework confers stability and protease protection to genetically fused cargo proteins^21,30,31^. Thus, we hypothesized that it might exert similar beneficial effects to the yCD proteins. Wild-type and the mutant yCDs were genetically fused to Cry3Aa to generate the corresponding fusion crystals (Supplementary Fig. 2B), and their enzymatic activities were determined. All the Cry3Aa fusion protein crystals remained active against 5-FC, albeit at a slightly lower conversion efficiency compared with their free protein counterparts (Figs. 3A and 1A), with the Cry3Aa-yCD fusion crystals exhibiting the most pronounced decrease in activity. Given the conformational difference at the C-terminus as revealed by the yCD-M100H’s structure, it is likely that immobilization of the enzyme affects the conformation of wild-type yCD to a larger extent than yCD-M100H, keeping it in a lesser active conformation. As with its soluble counterpart, Cry3Aa-yCD-M100H remained the most efficient, achieving 100% 5-FU conversion within 5 min (Fig. 3A, green). In addition, Cry3Aa-yCD-M100H exhibited the longest half-life among all the Cry3Aa-yCD variants tested. While Cry3Aa-yCD-M100H retained over 50% activity after 48 h incubation at 37 °C and 20% after 14-day incubation (Fig. 3B, green), all other constructs exhibited significantly shorter half-life, displaying < 40% activity and nearly no activity after 48-h and 14-day incubation, respectively (Fig. 3B). We, therefore, decided to focus on Cry3Aa-yCD-M100H for the subsequent investigations.

It has been shown that particle size, shape, and surface charge affect cellular uptake^32^. The morphology of Cry3Aa-yCD-M100H particles was therefore examined prior to testing their uptake efficiency into macrophages. Scanning electron microscopy (SEM) revealed that the fusion crystals were rod-shaped particles (Fig. 3C) with an average size ~810 nm (PDI: 0.117) based on dynamic light scattering, and were slightly negatively charged (zeta potential: −7.02 mV) (Fig. 3D, E). These measurements were similar to those observed for the native Cry3Aa crystals^33^.

### Cellular uptake and cytotoxicity of Cry3Aa-yCD-M100H crystals

**Fig. 4:**
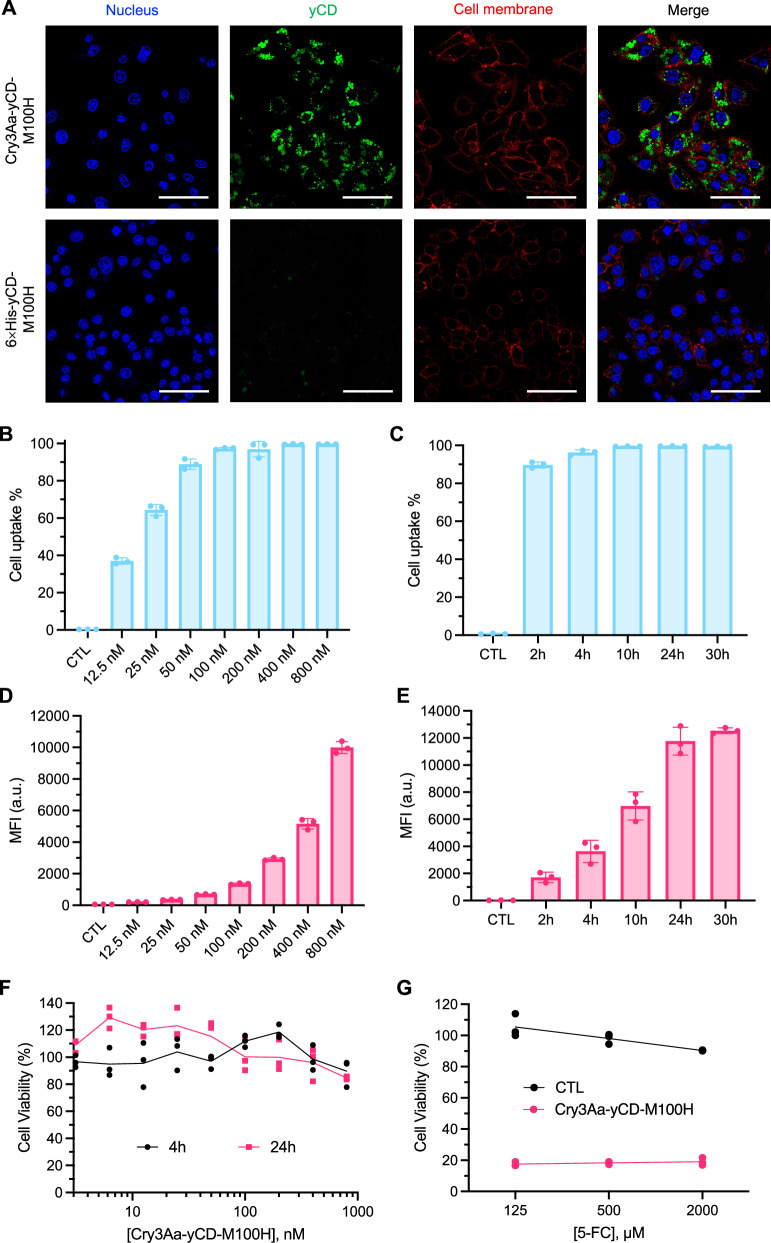
Cellular uptake efficiency and cytotoxicity of Cry3Aa-yCD-M100H in macrophages. **A** Confocal micrographs of RAW 264.7 cells treated with 50 nM Alexa-488-labeled Cry3Aa-yCD-M100H protein crystals (upper panel, green) or Alexa-488-labeled 6 × His-yCD-M100H protein (lower panel, green) for 24 h. Cells were co-stained with Hoechst 33342 (blue, nucleus), Wheat Germ Agglutinin Conjugate (red, cell membrane). Scale bar, 50 μm. **B**–**E** Quantitation of Alexa-488-labeled Cry3Aa-yCD-M100H internalized into RAW 264.7 cells by flow cytometry. **B**, **D** Concentration-dependent uptake of Cry3Aa-yCD-M100H was tested at various concentrations (12.5, 25, 50, 100, 200, 400, 800 nM) with a fixed 4-h incubation time at 37 °C. **C**, **E** Time-dependent uptake of 800 nM Cry3Aa-yCD-M100H into RAW 264.7 cells incubated for different incubation periods at 37 °C. All experiments in B-E were performed in triplicate and data were shown as mean ± SD. **F** Cytotoxicity of Cry3Aa-yCD-M100H to macrophages. RAW 264.7 cells were treated with different concentrations of Cry3Aa-yCD-M100H crystals for 4 or 24 h. Cell viability was evaluated by MTT at the end of the treatment period (*n* = 3). **G** Enzymatic activity and cytotoxicity effect of Cry3Aa-yCD-M100H encapsulated inside macrophages in catalyzing 5-FC to 5-FU (*n* = 3). Macrophages were incubated with 800 nM of Cry3Aa-yCD-M100H crystals for 24 h. Different concentrations of 5-FC were then added to the preloaded macrophages (MF/Cry3Aa-yCD-M100H).

Efficient uptake of therapeutics and their long-term retention and stability in the cells are important considerations when using macrophages as carriers for therapeutics. Previous studies from our laboratory have shown that Cry3Aa crystals could be efficiently taken up by macrophages but poorly by non-phagocytic cells^33^. To confirm that the fusion of yCD-M100H did not change the uptake property of Cry3Aa, the cellular uptake of Cry3Aa-yCD-M100H fusion crystals into RAW 264.7 and non-phagocytic cancer cells was evaluated. Confocal microscopy studies showed that the Alexa-488-labeled Cry3Aa-yCD-M100H crystals—but not the Alexa-488-labeled yCD-M100H protein - were readily internalized into RAW264.7 as bright green punctate dots could be observed in nearly all macrophages after 24 h (Fig. 4A), whereas scant signal could be detected in the treated cancer cells (Supplementary Fig. 4). Furthermore, the cellular uptake was both concentration- and time-dependent as indicated by the increasing number of fluorescent cells and mean fluorescence intensity (MFI) with increasing concentrations and lengths of incubation (Fig. 4B–E). At the low concentration of 100 nM, all the treated macrophages had taken up some labeled Cry3Aa-yCD-M100H within 4 h (Fig. 4B), while at 800 nM, significantly more internalized labeled crystals were observed in individual cells, reaching a plateau at 24 h (Fig. 4E).

Since the toxicity of the therapeutic cargo to the carrier cells is one major concern in cell-mediated delivery, the cytotoxicity of Cry3Aa-yCD-M100H on RAW 264.7 cells was investigated. In the absence of 5-FC, the Cry3Aa-yCD-M100H fusion crystals alone were non-toxic to the macrophages as minimal cell death was observed even after 24 h treatment at the highest concentration tested (800 nM) (Fig. 4F). Microscopic examination of these treated macrophages loaded with Cry3Aa-yCD-M100H fusion crystals (MF/Cry3Aa-yCD-M100H) showed similar morphology as that of the untreated (control) macrophages (Supplementary Fig. 5). We then incubated the MF/Cry3Aa-yCD-M100H with 5-FC to ensure that the observed non-toxic effect was not due to the degradation of the encapsulated Cry3Aa-yCD-M100H by intracellular proteases or other factors. Since only active Cry3Aa-yCD-M100H could catalyze the conversion of nontoxic 5-FC (Supplementary Fig. 6A) to toxic 5-FU, the number of viable macrophages after addition of 5-FC could also be used to correlate the enzymatic activity of the encapsulated fusion crystals. The killing of the RAW 264.7 was efficient as cell viability readily dropped to < 20% even at the lowest tested concentration of 125 μM 5-FC (Fig. 4G), confirming that the Cry3Aa-yCD-M100H remained active inside the host cells.

### Cytotoxic effect of Cry3Aa-yCD-M100H-loaded macrophages on cancer cells

**Fig. 5:**
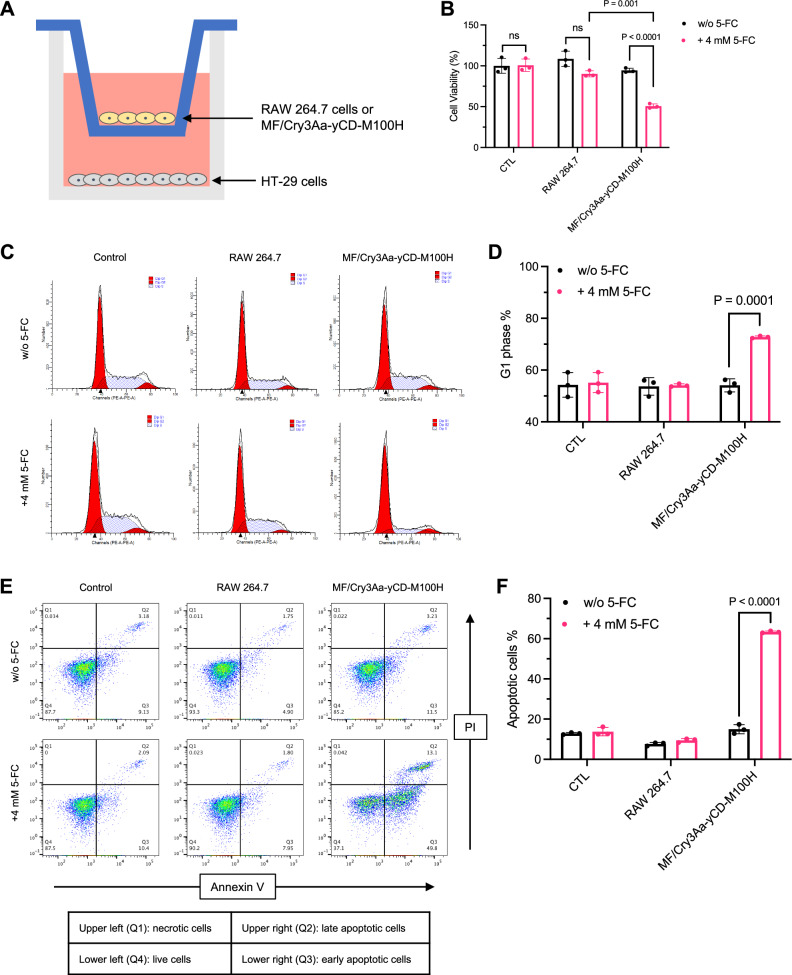
Anti-cancer effect of MF/Cry3Aa-yCD-M100H with 5-FC on HT-29 cells. **A** Schematic representation of the transwell set-up used in the investigation of the effect of MF/Cry3Aa-yCD-M100H in combination with 5-FC on HT-29. **B** Cytotoxicity of MF/Cry3Aa-yCD-M100H in the presence of 5-FC against HT-29 cancer cells. **C**–**F** Flow cytometric analysis of the HT-29 cells after treatment with the conditioned medium of MF/Cry3Aa-yCD-M100H or crystal-free MFs in the presence or absence of 5-FC. Histogram of the DNA content at different phases of cell cycle (**C**) and the percentage of HT-29 cells in G1 phase (**D**). Representative scatter plots of the differentially treated HT-29 cells stained with Annexin V and propidium iodide (PI) (**E**) and the percentage of apoptotic cells (**F**). All experiments were performed in triplicate. Data were shown as mean ± SD.

We then proceeded to evaluate the anti-cancer effect of MF/Cry3Aa-yCD-M100H in combination with 5-FC against the human colon cancer cells HT-29 (Fig. 5A) that were sensitive to 5-FU treatment (Supplementary Fig. 6B). MF/Cry3Aa-yCD-M100H in the presence of 5-FC exhibited significant anti-cancer effect, killing 50% of the HT-29 cells, which could be attributed to the conversion of 5-FC to 5-FU, as minimal killing was observed for the crystal-free RAW264.7 treatment group even in the presence of 5-FC (Fig. 5B). This result was further corroborated by flow cytometric analysis indicating that the combination of MF/Cry3Aa-yCD-M100H and 5-FC treatment could induce G0/G1 cell cycle arrest of the treated HT-29 cells (Fig. 5C, D and Supplementary Fig. 7) as well as apoptotic cell death as indicated by the detection of ~63% of apoptotic cells (Fig. 5E, F). Neither effect was observed for the HT-29 cells in the control group nor those exposed to the crystal-free macrophages. These results further confirmed the enzymatic activity of the encapsulated Cry3Aa-yCD-M100H and its use as an anti-cancer prodrug 5-FC-activating agent.

### Effect of Cry3Aa-yCD-M100H on tumor-targeting ability of macrophage

**Fig. 6:**
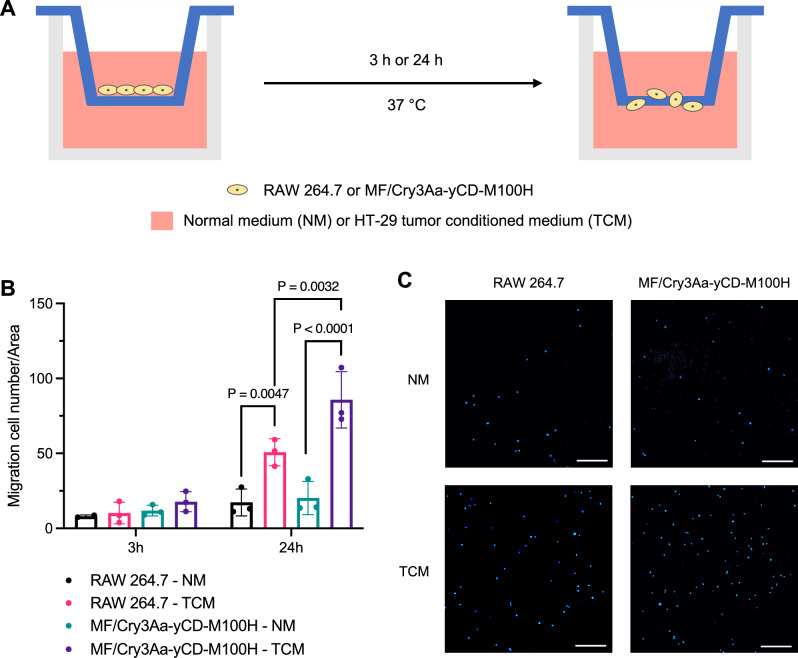
Targeting ability of MF/Cry3Aa-yCD-M100H. **A** Schematic illustration of the transwell assay used to examine the migratory response of MF/Cry3Aa-yCD-M100H or crystal-free macrophages towards the HT-29 conditioned culture medium in the lower chamber. NM normal medium, TCM tumor conditioned medium. **B** Quantification of macrophages migrated to the basolateral side in response to different media. For each sample, ten randomly-selected views of membrane were recorded, and the migration cells were counted by Fiji software. The experiments were performed in triplicate and in two independent trials. Representative result was presented and data were shown as mean ± SD. **C** Representative fluorescence images of the basolateral membranes of the transwell inserts after 24 h. Macrophages were stained with Hoechst 33342. Scale bar, 100 μm.

Tumor cells secrete chemokines, which naturally recruit monocyte/macrophage to tumor sites^34^. To ascertain whether the encapsulation of Cry3Aa-yCD-M100H affected the migration ability of RAW264.7, a transwell migration assay (Fig. 6A) was conducted. No migration was observed for any experimental groups in the normal medium (NM) even after 24-h incubation. In contrast, significant numbers of crystal-free or crystal-loaded macrophages were observed to have migrated from the apical side of the transwell insert to the basolateral side after 24 h, which was oriented toward the HT-29 tumor conditioned medium (TCM) in the bottom chamber (Fig. 6B, C). The macrophage migration towards the TCM was time-dependent, as there was no meaningful difference in the number of macrophages in the basolateral compartment between the different experimental groups at the earlier 3-h time point (Fig. 6B). These results indicated that the loading of Cry3Aa-yCD-M100H did not compromise the chemotactic ability of the carrier macrophages, which is essential for tumor-targeting.

### Tumor penetration and anti-tumor effect of MF/Cry3Aa-yCD-M100H in colon cancer spheroid

**Fig. 7:**
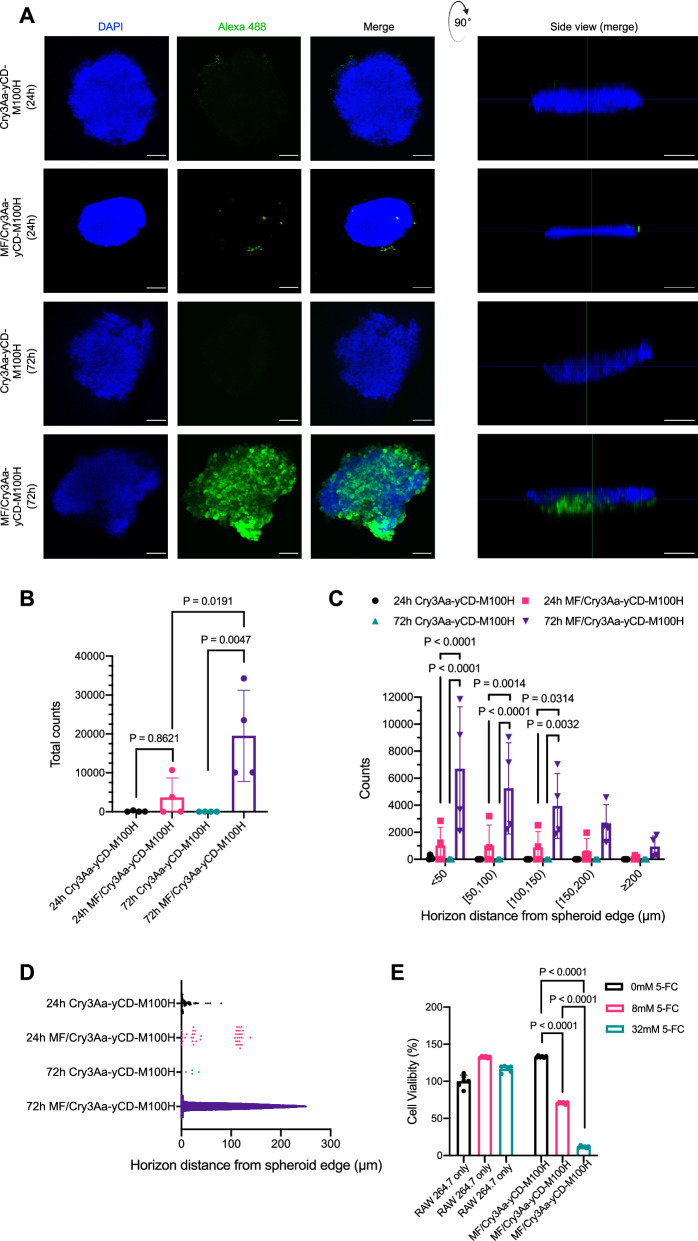
Penetration of MF/Cry3Aa-yCD-M100H and its anti-tumor efficacy in tumor spheroid*.* **A** Confocal images of HT-29 spheroids treated with Cry3Aa-yCD-M100H or MF/Cry3Aa-yCD-M100H for 24 or 72 h. Images shown are horizontal (left, scale bar: 100 μm) and vertical (right, scale bar: 75 μm) cross sections of the middle layers of spheroids. Cry3Aa-yCD-M100H were labeled with Alexa-488 maleimide dye (green) and HT-29 spheroids were stained with DAPI (blue). **B**–**E** Quantitative analysis of the crystals (**B**) in the middle layers of the tumor spheroid and (**C**, **D**) their distributions in different penetration distance zones. The penetration depth was measured horizontally from spheroid edge. Image analysis was performed using CellProfiler. Data were shown as mean ± SD (*n* = 4).

It has been shown that 3D cultures provide models that better mimic conditions in vivo^35,36^. As such, HT-29 spheroids were generated to evaluate the penetration ability of MF/Cry3Aa-yCD-M100H. Confocal microscopy analysis indicated that the Alexa488-labeled MF/Cry3Aa-yCD-M100H-488 could penetrate deep into the HT-29 tumor spheroids after 72-h incubation, whereas no green fluorescence signal could be detected for the Cry3Aa-yCD-M100H-treated HT-29 spheroids (Fig. 7A). Notably, the tumor penetration was time-dependent. At 24-h, most MF/Cry3Aa-yCD-M100H were predominantly located on the periphery, clustering around the cell membranes (Fig. 7A). But after 72-h incubation, abundant green fluorescence signals could be observed throughout the spheroid, including the inner core, with approximately 20% of the MF/Alexa-labeled Cry3Aa-yCD-M100H found in >150 µm layers, indicating the successful penetration and accumulation of the fusion crystals inside the tumor spheroid mediated by the macrophages (Fig. 7A–D, and Supplementary Fig. 8–9). On the other hand, the penetration and accumulation of the Cry3Aa-yCD-M100H only group was much less, and the crystals were mostly trapped at the periphery even after the extended period of incubation. Thus, the encapsulation of the Cry3Aa-yCD-100H fusion crystals in macrophages significantly enhances the penetration ability of Cry3Aa-yCD-100H fusion crystals.

We then proceeded to investigate whether these improved attributes could be translated to enhanced cancer killing in the spheroids. HT-29 spheroids were treated with crystal-free macrophages or MF/Cry3Aa-yCD-M100H in combination with 5-FC. As shown in Fig. 7E, MF/Cry3Aa-yCD-M100H and 5-FC were effective against the tumor spheroids, as significant reduction in the number of viable HT-29 cancer cells could be observed at the end of the treatment period. Collectively, these results further affirm the intracellular stability of Cry3Aa-yCD-M100H and its suitability for use in the macrophage-mediated delivery of the prodrug-activating enzyme cytosine deaminase for yCD/5-FC cancer therapy.

## Conclusion

In this study, our analysis of the yeast CD structure led to the identification of a cavity situated in close proximity to the active site harboring two cysteines and a histidine, which we hypothesized allows the easy access of surrounding bulk solution to one of the active site cysteines, Cys91. By substituting Met100 at the active site entry with the bulkier histidine, the solvent exposed surface area of Cys91 was reduced, as validated by X-ray crystallographic study on the resultant yCD-M100H mutant. This mutant exhibited much improved lifetime and enzymatic activity at physiological temperature compared with the wild-type yCD and the previously reported triple mutant yCD developed via computational design^9^. To further enhance the stability of yCD-M100H, we fused it to Cry3Aa to generate Cry3Aa-yCD-M100H. The Cry3Aa crystal framework confers excellent protection to yCD-M100H against proteolytic degradation, and enables it to be encapsulated inside macrophages (MF/Cry3Aa-yCD-M100H) while retaining its catalytic activity. MF/Cry3Aa-yCD-M100H were shown to be able to penetrate deep into the HT-29 spheroid and effectively kill cancer cells in the presence of prodrug 5-FC.

As demonstrated in this study, Cry3Aa crystal platform is well-suited for use as a stabilizer of macro-biomolecule for cell-mediated therapeutic delivery, given its biocompatible, non-toxic, and excellent cargo protection properties. And considering the better safety profile of prodrug, this platform can potentially be applied to other prodrug systems.

## Materials and methods

### Cell culture

Human colorectal carcinoma HT-29 cells and mouse breast cancer cells 4T1 were gifts from Prof. Thomas Y. C. Leung (The Hong Kong Polytechnic University, HKSAR), and mouse leukemic monocyte macrophage RAW 264.7 cells from Dr. Daniel Lee (The Hong Kong Polytechnic University, HKSAR). HT-29 cells and 4T1 cells were cultured in RPMI-1640 medium (Gibco, #23400021) while RAW 264.7 cells were maintained in DMEM medium (Gibco, 11960044). All media were supplemented with 10% fetal bovine serum (FBS) (Hyclone, #SV30160.03) and 1% penicillin-streptomycin (P/S) (Hyclone, #SV30010) unless otherwise indicated. Cells were incubated at 37 °C and 5% CO_2_ in a humidified chamber.

### Construction and expression of yeast CD protein and its mutants

*fcy1* encoding *Saccharomyces cerevisiae* cytosine deaminase (yCD) was amplified from plasmid pPH37, which was a gift from John McCusker^37^, and the resultant amplicons were subcloned into the *NdeI* and *XhoI* sites in pET28a. Mutants of yCD were generated by site-directed mutagenesis (Kapa Biosystems) performed on the aforementioned yCD-containing pET28a plasmid. Resultant plasmids were transformed into *E. coli* BL21 (DE3) and protein expression was induced with 0.5 mM IPTG at 16 °C for 20 h. Purified 6x his-tagged proteins were obtained by nickel affinity chromatography and dialyzed into 20 mM Tris, 300 mM NaCl, pH 7.5 buffer to remove imidazole. DNA integrity was verified by DNA sequencing (BGI), and protein purity was verified by SDS-PAGE.

### Construction and expression of Cry3Aa-yCD protein crystal and its mutants

*fcy1* and its mutated variants were cloned downstream of *cry3Aa* in the *cry3Aa*-containing pHT315 plasmid to generate pHT315-Cry3Aa-yCD and its variants. The plasmids were electroporated into *Spo-Bt* strain 407-OA for expression of the fusion crystals at 25 °C for 48 h. Cells were harvested and lysed, and the fusion crystals were isolated by sucrose gradient centrifugation followed by extensive washing with ddH_2_O. The purity was checked by SDS-PAGE.

### Enzymatic activity and kinetic stability assays

5-fluorocytosine (5-FC, TCI America, #F0321) and 5-fluorouracil (5-FU, TCI America, #F0151) have an absorbance peak at 276 nm and 266 nm, respectively. The enzymatic activity of the yCD proteins and their fusion crystal counterparts was determined by measuring the conversion of 5-FC to 5-FU based on their corresponding absorbance at 276 nm and 266 nm. Briefly, 5-FC and 5-FU were dissolved in 1× PBS and DMSO. To generate a calibration curve, a series of 5-FC, 5-FU alone at different concentrations, and 5-FC/5-FU mixtures mixed at different molar ratios in PBS were prepared, and their absorbance from 266 nm to 276 nm (266, 268, 270, 272, 274, 276 nm) was measured and plotted. The percentage of 5-FU in the reaction mixture was derived using the following equation:

abs _mixture_ = abs _5-FU_ × 5-FU% + abs _5-FC_ × (1 – 5-FU%)

To determine the enzymatic activity of the yCD proteins, 1 mM 5-FC was added to 250 nM wild-type or mutant 6x his-tagged yCD protein in PBS and reacted at 37 °C, and the absorbance was monitored and recorded on a Tecan Infinity® M1000 Pro plate reader. The production of 5-FU was determined by measuring the absorbance at 266 nm and 276 nm and derived as described above. The enzymatic activities of the Cry3Aa fusion crystals were evaluated similarly, except in this case, the yCD proteins were replaced with their corresponding fusion crystal counterparts, and 500 nM concentration was used instead.

The kinetic stability of the yCD fusion protein crystals was evaluated by pre-treating 20 μM of the fusion crystals in PBS at 37 °C for different lengths of time (2, 4, 14 days), and then determined their residual enzymatic activities following the same procedure as described above. The percentage of residual activity was normalized to the activity of the corresponding fusion crystals without pretreatment.

### Thermal stability studies

The thermal stability of the purified yCD proteins was determined using circular dichroism spectroscopy. Briefly, 0.1 mg/mL of the purified 6x his-tagged yCD proteins in 10 mM sodium phosphate buffer (pH 7.4) were loaded into a rectangular quartz cell (0.1 cm path length), and the yCD signals at 220 nm were monitored and recorded with increasing temperature from 25 °C to 85 °C with increments of 1–5 °C on a J-810 spectropolarimeter (Jasco, Tokyo, Japan). The reading at 25 °C was considered as 100% protein at folded state, while 0% folded at 85 °C. Data was plotted against temperature, and the melting temperature (*T*_m_) was calculated at which 50% of the protein remained folded by GraphPad Prism software (10.1.1).

### Enzyme oxidation studies

Five hundred nanomole wild-type yCD and mutant yCD-M100H were pre-incubated in PBS with hydrogen peroxide (Scharlau) in concentrations ranging from 7.84 mM to 4.90 M for 10 min. Catalase (~500 units/mL; Sigma-Aldrich, #C9322) was added to remove the residual H_2_O_2_. The enzymatic activities were then determined using 5-FC, and the percentage of enzyme activities was normalized to the corresponding enzymes without hydrogen peroxide treatment.

### Crystallization and data collection of yCD-M100H

The purified 6×His-yCD-M100H protein was subjected to overnight thrombin (Sigma-Aldrich, #T4648) digestion at room temperature to remove the 6x his-tag. After cleavage, the his-tag was removed and the protein was concentrated to 10 mg/mL in PBS using an Amicon MWCO 3k concentrator (Merck Millipore, #UFC500396).

Crystallization was set up by the method of hanging drop vapor diffusion at 4 °C^13^. Two microliters of protein solution was mixed with 1 μL of reservoir solution consisting 0.1 M sodium cacodylate, 0.16 M–0.20 M sodium acetate, and 20%–30% PEG 8000. The final protein crystal was grown in 0.1 M sodium cacodylate, 0.16 M sodium acetate, and 24% PEG 8000, and cryo-cooled in 30% glycerol prior to data collection.

The diffraction data for the yCD-M100H were collected from crystals cooled to 277.15 K on microfocus beamline 05A at a wavelength of 0.99984 Å using a RAYONIX MX-300 HS detector at NSRRC, Taiwan. Data processing and scaling were performed with HKL2000^38^.

### Phasing and refinement of yCD-M100H

The yCD-M100H structure was determined using PHASER^39^ with the wild-type yCD structure (PDB ID: 1OX7) as searching model. Model building and refinement of the yCD-M100H structure were carried out using the programs Refmac5^40^ and Coot^41^. Web server PDB_REDO was used to generate optimal restraint settings for refining the yCD-M100H structure^42^. The coordinate and structure factor have been deposited to the Protein Data Bank (PDB ID: 9K1K). 97.97% of the residues were in the favored regions, 2.03% in the allowed regions, and 0% in the outlier regions of the Ramachandran plot.

### Kinetic measurements

The enzymatic activity of the wild-type 6x His-yCD and the mutant 6x His-yCD-M100H was determined by measuring the conversion of 5-FC to 5-FU based on the UV spectroscopic measurements at absorbance 266 nm and 290 nm, respectively. A calibration curve was first established by measuring the UV absorbance at the indicated wavelength for different concentrations of 5-FC and 5-FU, and which were used to derive the following formula:

$$[5-{{\rm{FU}}}]=\frac{4.0481\times {{\rm{Abs}}}_{290}\,-2.6565 \, {{\rm{x}}}\; {{\rm{Abs}}}_{266}}{4.0481\times 1.3035-2.6565\,\times 5.2849}$$

The activity of the respective proteins was then determined by incubating 500 nM proteins in PBS at 37 °C with a range of 5-FC concentrations for different lengths of time, and the corresponding absorbance at the indicated wavelength was recorded. Initial velocity was calculated as a function of the intial slope by curve fitting the resulting plot. *K*_m_, *V*_max_, and *k*_cat_ were determined from a Lineweaver-Burke plot of the resulting data.

### DLS and zeta potential measurements of Cry3Aa-yCD-M100H crystals

Cry3Aa-yCD-M100H crystals were resuspended in 1× PBS to 50 μg/mL. The particle size and zeta potential measurements were conducted using Malvern Zetasizer Nano ZS90 (Malvern Instruments Ltd., Malvern, UK) at 25 °C.

### Scanning electron microscopy of Cry3Aa-yCD-M100H crystals

Protein crystals were resuspended in ddH_2_O to a final concentration of 0.4 mg/mL. Two microliters of the suspension were added to a copper stub and air-dried. Following gold coating by a Sputter Coater S150B (Edwards), the sample was imaged using a SU8000 (Hitachi) scanning electron microscope operated at 5 kV and a working distance of 8.4 mm.

### Cellular uptake of Cry3Aa-yCD-M100H crystals

The uptake efficiency of Cry3Aa-yCD-M100H crystals and the purified 6×His-yCD-M100H protein into RAW264.7 was evaluated by labeling the fusion crystal and soluble protein with Alexa Fluor™ 488 C_5_ Maleimide dye (Thermo Fisher Scientific, #A10254) following manufacturer’s instruction. Excess dye was removed from Cry3Aa-yCD-M100H crystals by repeated washing with ddH_2_O following by centrifugation, while spin desalting columns were used for the 6×His-yCD-M100H protein. The purified labeled fusion crystals and proteins were then used for the subsequent confocal microscopy studies.

2 × 10^4^ RAW 264.7 cells were seeded on a 35 mm confocal dish (MatTek, #P35G-1.5-14-C). Fifty nanomole of the Alexa-488-labeled Cry3Aa-yCD-M100H crystals or Alexa-488-labeled 6×His-yCD-M100H protein were then added for 24-h incubation at 37 °C/5% CO_2_. After washing cells with 20 U/mL heparin (Sigma-Aldrich, #H0393) in PBS to remove noninternalized crystals/protein, cells were stained with Hoechst 33342 (Thermo Fisher Scientific, #H1399) and Wheat Germ Agglutinin conjugated with Alexa-647 (Thermo Fisher Scientific, #W32466) for labeling the nuclei and cell membranes, respectively. Images were obtained using a Leica SP8 confocal microscope.

The confocal microscopy analysis of the cellular uptake of Cry3Aa-yCD-M100H into the non-phagocytic HT-29 and murine breast cancer 4T1 cells followed the same aforementioned procedure, except in this case, 1 × 10^4^ cancer cells were seeded on the confocal dish.

Flow cytometric analysis was employed to quantitate the loading efficiency of Cry3Aa-yCD-M100H crystals into macrophages. In brief, 1 × 10^5^ RAW 264.7 cells seeded in a 24-well plate were treated with either different concentrations (12.5 - 800 nM) Alexa-488-labeled Cry3Aa-yCD-M100H crystals for 4 h for concentration-dependent studies or 100 nM Alexa-488-labeled Cry3Aa-yCD-M100H crystals incubated for different lengths of time (0.5, 1, 2, 4, 6, and 8 h) for time-dependent studies at 37 °C/5% CO_2_. At the end of the experiments, cells were washed with 20 U/mL heparin in PBS and harvested using 5 mM EDTA in PBS. Detected cells were then rinsed with cold PBS and applied to a BD FACSVerse flow cytometer (Supplementary Fig. 10A). Data were collected (BD FACSuite software (1.0.6)) and analyzed using FlowJo (10.4).

### Cytotoxicity of Cry3Aa-yCD-M100H on macrophages

RAW 264.7 cells were seeded at 1 × 10^4^ cells/well in a 96-well plate and incubated overnight at 37 °C/5% CO_2_ to allow for adherence. A series of concentrations of Cry3Aa-yCD-M100H crystals was then added to the adhered cells and incubated for another 4 or 24 h. At the end of treatment, the cells were washed with PBS to get rid of noninternalized crystals and further incubated for another 24 h before the determination of cell viability by MTT (3-(4,5-dimethylthiazol-2-yl)-2,5-diphenyltetrazolium bromide) assay. Briefly, 10 μL of 5 mg/mL MTT was added following 4-h incubation. Formazan produced was dissolved in DMSO, and the absorbance at 565 nm was measured by a Tecan infinity® M1000 pro plate reader. The % of cell viability was plotted against crystal concentration using GraphPad Prism software (10.1.1).

To evaluate whether uptake of Cry3Aa-yCD-M100H crystals induced any morphology changes to RAW 264.7 cells, Cry3Aa-yCD-M100H-internalized macrophages were prepared by incubating the cells with 800 nM of the fusion crystals for 4 h, then imaged on a Nikon TE300 fluorescence microscope. Untreated cells were set up in parallel for comparison.

### Enzymatic activity of Cry3Aa-yCD-M100H crystals encapsulated in macrophages

The cell viability of macrophages was utilized to assess the enzymatic activity of the encapsulated Cry3Aa-yCD-M100H fusion crystals. Briefly, RAW 264.7 macrophages were incubated with 800 nM Cry3Aa-yCD-M100H crystals for 24 h. At the end of incubation, the Cry3Aa-yCD-M100H-loaded macrophages were detached using 5 mM EDTA in PBS. 5000 of these macrophages were then seeded onto a 96-well plate and treated with various concentrations of 5-FC for 72 h. The cell viability of the treated cells were measured by MTT assay.

### In vitro anti-cancer activity of MF/Cry3Aa-yCD-M100H with 5-FC

The effect of 5-FC or 5-FU on RAW 264.7 or HT-29 cells was first conducted to confirm their cytotoxicity using MTT reagent. In brief, seeded RAW 264.7 (5000 cells/well) or HT-29 (2500 cells/well) were treated with 5-FC or 5-FU at a series of concentrations for 72 h. The cell viability was then determined by MTT assay.

The anti-cancer effect of Cry3Aa-yCD-M100H-loaded RAW 264.7 (MF/Cry3Aa-yCD-M100H) in combination with 5-FC was evaluated on the human colon cancer cells HT-29 using a transwell system. 1 × 10^4^ HT-29 cells were seeded in a 24-well plate with or without 4 mM 5-FC in RPMI medium. MF/Cry3Aa-yCD-M100H were prepared by incubating 800 nM Cry3Aa-yCD-M100H with RAW 264.7 for 24 h. 5 × 10^4^ of the crystal-loaded cells were added to the top chamber of the transwell insert. Cells were incubated for 3 days, after which the transwell inserts were removed and the cell viability of HT-29 was determined by MTT assay.

### Cell cycle arrest and apoptosis

MF/Cry3Aa-yCD-M100H and crystal-free macrophages were incubated with or without 5-FC (4 mM) for 24 h. The conditioned culture media were collected and 2-fold diluted using fresh culture medium for the subsequent cell cycle and apoptosis experiments.

For cell cycle assays, HT-29 cells seeded at a density of 1 × 10^5^ cells/well in a 12-well plate were incubated in serum-free medium for 24 h for cell synchronization. The cells were then treated with the conditioned media or fresh media for 24 h. At the end of treatment, cells were trypsinized, fixed in 70% ethanol for 45 min on ice, and then stained with 10 μg/mL propidium iodide (PI, Sigma-Aldrich, #81845) in the presence of Triton X-100 (Macklin, #X768613) and RNase for 30 min in dark before subjected to flow cytometric analysis (Supplementary Fig. 10B). Data were analyzed using ModFitLT (5.0.9).

For apoptosis assays, HT-29 cells seeded in a 12-well plate were treated with the conditioned or fresh media for 24 h. Cells were trypsinized, fixed in ethanol, and then stained with annexin V (BioLegend, #640906) and PI following manufacturer’s instruction (BioLegend). Cells were analyzed on a BD FACSVerse flow cytometer (Supplementary Fig. 10C). Data were analyzed using FlowJo (10.4).

### In vitro transwell migration assay

HT-29 conditioned medium was prepared by incubating HT-29 in RPMI medium for 24 h. The culture medium was transferred into a conical Falcon tube and centrifuged for 10 min at 1500 rpm to remove any cells/debris. The supernatant (HT-29 tumor conditioned medium) collected was used for the transwell migration experiment.

10,000 RAW 264.7 or MF/Cry3Aa-yCD-M100H were seeded in a transwell insert (SPL, #SPL-35224) in serum-free culture medium supplemented with 0.2% BSA and placed into a 24-well plate containing either control or HT-29 tumor conditioned medium (TCM) in the lower chamber. After incubation at 37 °C for either 3 or 24 h, the cells on each transwell membrane were fixed by ethanol and rinsed with 1 × PBS. Cotton swabs were used to gently remove the cells on the upper compartment of the transwell membrane. The membranes were then excised and stained with Hoechst 33342 for examination under a Nikon TE300 fluorescence microscope. 10 views of each membrane were randomly selected and captured for cell enumeration by Fiji software (2.14.0)^43^.

### Tumor spheroid penetration and anti-tumor efficacy of MF/Cry3Aa-yCD-M100H

The penetration ability of MF/Cry3Aa-yCD-M100H and its anti-tumor efficacy in combination with 5-FC were evaluated against HT-29 tumor spheroids. Tumor spheroids were generated by culturing 5,000 HT-29 cells in RPMI medium supplemented with 10% FBS in a low attachment hydro 96-well plate (Thermo Fisher) at 37 °C for 96 h. The size of spheroids was around 400–500 nm in diameter.

For the tumor penetration studies, Cry3Aa-yCD-M100H crystals were labeled with Alexa Fluor™ 488 C_5_ Maleimide dye (Thermo Fisher). 100 nM of labeled Cry3Aa-yCD-M100H-488 or MF/Cry3Aa-yCD-M100H-488 (equivalent to 100 nM protein crystals) were then incubated with the HT-29 spheroids for 24 or 72 h. At the end of incubation, the treated spheroids were washed with 1 × PBS before transferring to a confocal dish. The spheroids were fixed with PFA, stained with 1 μg/mL of DAPI (TCI America, #D5888), and dehydrated using ethanol. The fixed spheroids were then incubated with 3D tissue clearing reagent (Corning, #5731) before confocal imaging on a Leica SP8. Images were analyzed using CellProfiler (4.2.5).

To evaluate the anti-tumor efficacy of MF/Cry3Aa-yCD-M100H in combination with 5-FC, HT-29 spheroids were treated with either MF/Cry3Aa-yCD-M100H (equivalent to 100 nM protein crystals) or crystal-free RAW264.7 (equivalent to the number of macrophages in MF/Cry3Aa-yCD-M100H) for 24 h. After incubation, 8 mM or 32 mM 5-FC was added to the spheroid for incubation for another 2 days. Here, higher concentrations of 5-FC were used owing to the more complex tumor environment in the spheroid, including the hypoxic core region known to be resistant to a variety of chemotherapeutics^44^. Cell viability was tested using the CellTiter-Lumi^TM^ Luminescent 3D II cell viability assay kit (Beyotime, #C0062L). Briefly, 100 μL of CellTiter-Lumi^TM^ Luminescent 3D detection reagent was added following 25-min incubation, the luminescence signal was measured on a Tecan Infinity^®^ M1000 Pro plate reader. The % of cell viability was plotted using GraphPad Prism software (10.1.1).

### Statistics and reproducibility

Data were presented as means ± standard deviations (SDs), and the number of independent biological replicates is noted in the figure legends where appropriate. For statistical significance analysis, one-way ANOVA followed by Tukey’s post hoc test was applied when there was only one independent variable, while two-way ANOVA was employed for experiments involving two independent variables to calculate the *p* values. The exact *p* values were displayed in the figures. All data calculations and statistical analyses were performed by GraphPad Prism software (10.1.1).

### Reporting summary

Further information on research design is available in the Nature Portfolio Reporting Summary linked to this article.

## Supplementary information

Supplementary information

Supplementary Data 1

Supplementary Data 2

Description of Additional Supplementary Materials

Reporting summary

## Data Availability

The structural information of yCD-M100H has been deposited in the Protein Data Bank under accession code 9K1K. Source data used to generate the main figures and tables in the manuscript are available in Supplementary Data 1, while those used for the figures and tables in the Supplementary Information as well as any primer sequences are provided in Supplementary Data 2.
