# Supplementary material for: An engineered yeast cytosine deaminase with improved catalytic activity and stability for macrophage-mediated enzyme/prodrug therapy
Source: Commun Biol. 2025 Nov 13;8:1562. doi: 10.1038/s42003-025-08931-x (PMC12615592; doi:10.1038/s42003-025-08931-x)
Supplement: Supplementary file 1 — Supplementary information [file 42003_2025_8931_MOESM1_ESM.pdf]

Supplementary information:

An engineered yeast cytosine deaminase with improved catalytic activity and stability for macrophage-mediated enzyme/prodrug therapy

Jiale Zheng[1#], Jiahao Zhou[1#], Kristen Wing Yu Yung[1], Qipeng Hu[1], Marianne M. Lee[1]*, Michael K. Chan[1]*.

[1]School of Life Sciences and Center of Novel Biomaterials, The Chinese University of Hong Kong, Shatin, Hong Kong SAR 999077, China.

[#]*These authors contributed equally: Jiale Zheng, Jiahao Zhou*

**Corresponding authors: M.M.L.: mariannemmlee@cuhk.edu.hk, M.K.C.: michaelkchan88@cuhk.edu.hk*

```
      yCD  MVTGGMASKWDQKGMDIAYEEAALGYKEGGVPIGGCLINNKDGSVLGRGHNMRFQKGSATLHGEISTLENCGRLEGKVY
   yCD-TM  MVTGGMASKWDQKGMDIAYEEALLGYKEGGVPIGGCLINNKDGSVLGRGHNMRFQKGSATLHGEISTLENCGRLEGKVY
yCD-M100H  MVTGGMASKWDQKGMDIAYEEAALGYKEGGVPIGGCLINNKDGSVLGRGHNMRFQKGSATLHGEISTLENCGRLEGKVY
yCD-M100L  MVTGGMASKWDQKGMDIAYEEAALGYKEGGVPIGGCLINNKDGSVLGRGHNMRFQKGSATLHGEISTLENCGRLEGKVY
  yCD-TMH  MVTGGMASKWDQKGMDIAYEEALLGYKEGGVPIGGCLINNKDGSVLGRGHNMRFQKGSATLHGEISTLENCGRLEGKVY
  yCD-TML  MVTGGMASKWDQKGMDIAYEEALLGYKEGGVPIGGCLINNKDGSVLGRGHNMRFQKGSATLHGEISTLENCGRLEGKVY
                    10        20        30        40        50        60        70

      yCD  KDTTLYTTLSPCDMCTGAIIMYGIPRCVVGENVNFKSKGEKYLQTRGHEVVVVDDERCKKIMKQFIDERPQDWFEDIGE
   yCD-TM  KDTTLYTTLSPCDMCTGAIIMYGIPRCVIGENVNFKSKGEKYLQTRGHEVVVVDDERCKKLMKQFIDERPQDWFEDIGE
yCD-M100H  KDTTLYTTLSPCDMCTGAIIHYGIPRCVVGENVNFKSKGEKYLQTRGHEVVVVDDERCKKIMKQFIDERPQDWFEDIGE
yCD-M100L  KDTTLYTTLSPCDMCTGAIILYGIPRCVVGENVNFKSKGEKYLQTRGHEVVVVDDERCKKIMKQFIDERPQDWFEDIGE
  yCD-TMH  KDTTLYTTLSPCDMCTGAIIHYGIPRCVIGENVNFKSKGEKYLQTRGHEVVVVDDERCKKLMKQFIDERPQDWFEDIGE
  yCD-TML  KDTTLYTTLSPCDMCTGAIILYGIPRCVIGENVNFKSKGEKYLQTRGHEVVVVDDERCKKLMKQFIDERPQDWFEDIGE
           80        90        100       110       120       130       140       150
```

Supplementary Fig. 1. Multiple protein sequence alignment of the yeast cytosine deaminase (yCD) from *Saccharomyces cerevisiae* and its mutants used in this study. Sites of mutations are highlighted, with the native residues shaded in yellow and the mutations in other colors.

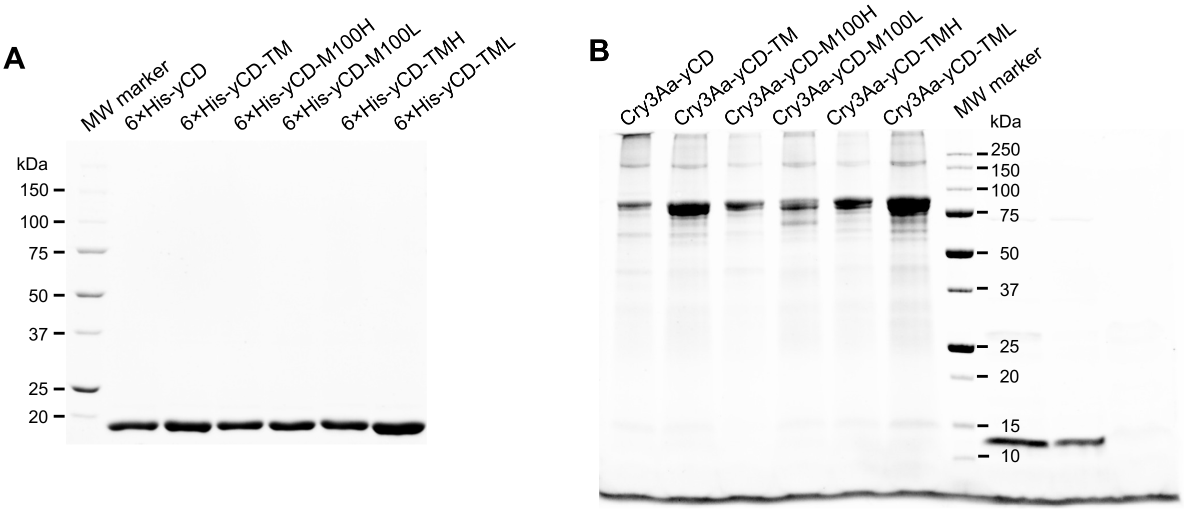

Supplementary Fig. 2. SDS-PAGE analyses of the wild-type and mutant yeast cytosine deaminase (A) free proteins and (B) the corresponding fusion protein crystals. Unscropped images were shown. The right three lanes in (B) were not related to this study.

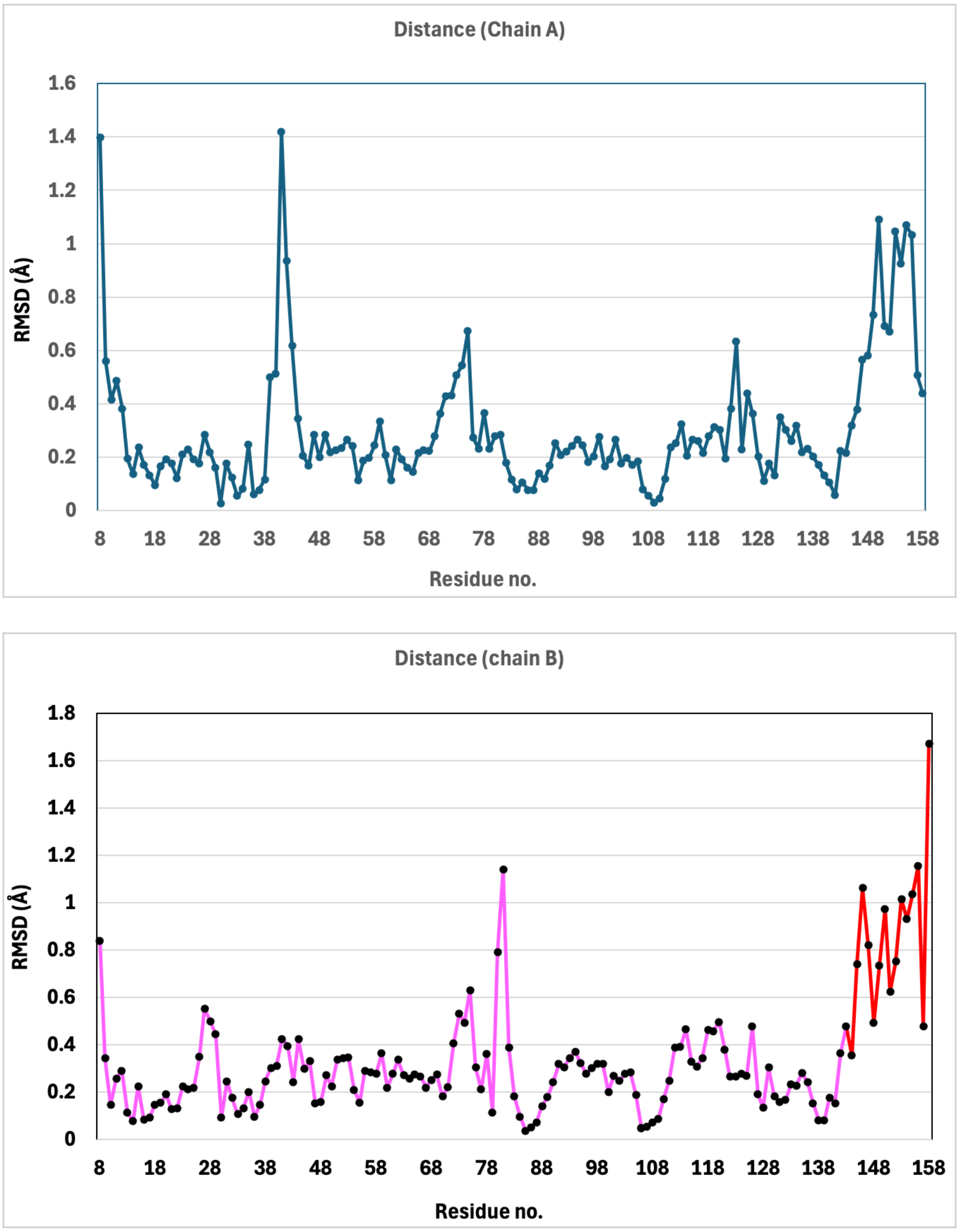

Supplementary Fig. 3. RMS analysis by the GESAMT program showing the deviation of α-carbon position of every residue between the yCD (PDB ID: IOX7) and yCD-M100H structures for each subunit. The colors used in the plots match those used for the yCD-M100H dimer in Fig. 2.

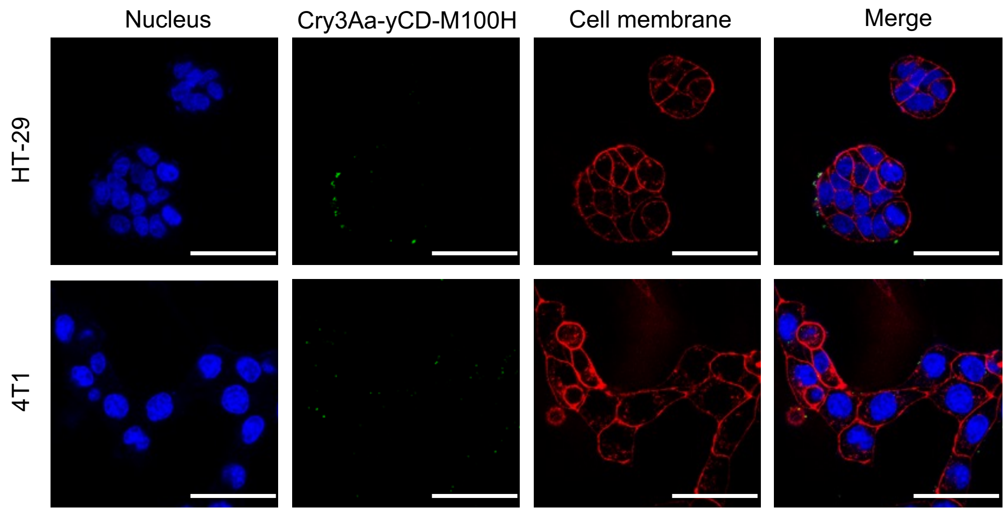

Supplementary Fig. 4. Cellular uptake of Cry3Aa-yCD-M100H in HT-29 and 4T1 cells. Representative confocal micrographs of HT-29 (upper panel) and 4T1 cells (lower panel) treated with 50 nM Alexa-488-labeled Cry3Aa-yCD-M100H protein crystals for 24 h. Cells were co-stained with Hoechst 33342 (blue, nucleus), Wheat Germ Agglutinin Conjugate (red, cell membrane). Scale bar, 50 µm.

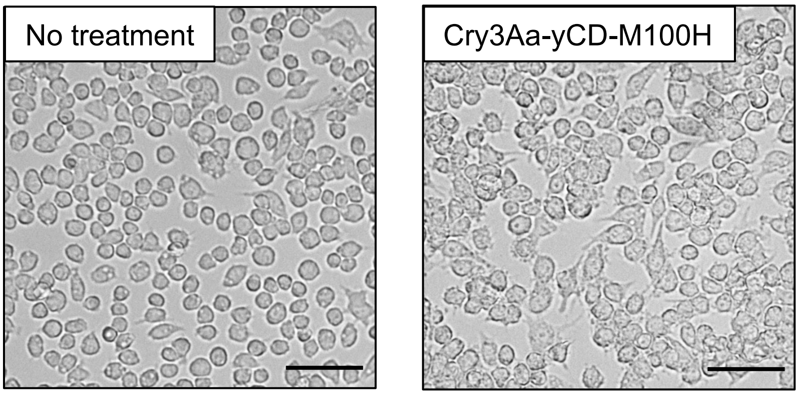

Supplementary Fig. 5. Representative brightfield microscopy images of RAW 264.7 cells without (left) or with (right) Cry3Aa-yCD-M100H loading (800 nM, 4h). Scale bar, 50 µm.

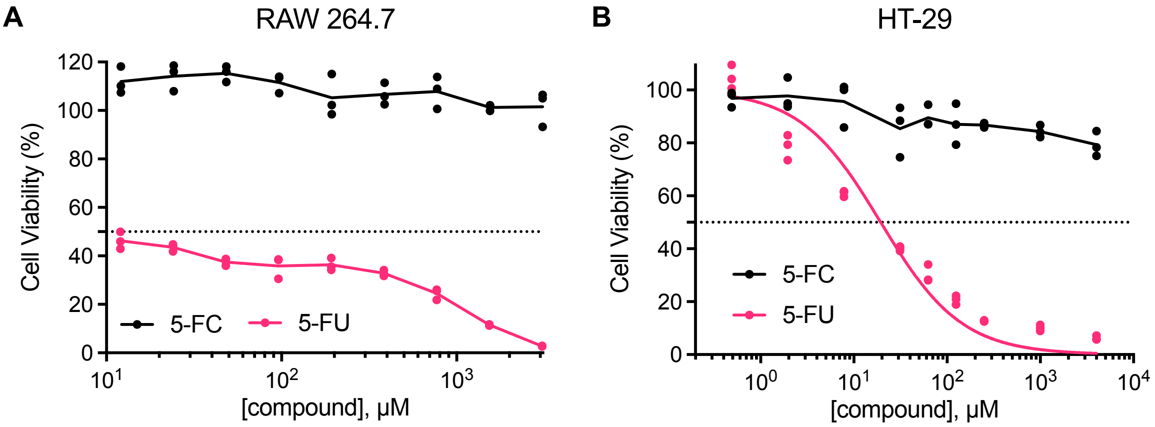

Supplementary Fig. 6. Cytotoxicity of 5-FC and 5-FU against RAW 264.7 (A) and HT-29 (B). Cells were treated with a series of concentrations of 5-FC or 5-FU for 3 days (n = 3). Cell viability was measured by MTT reagent.

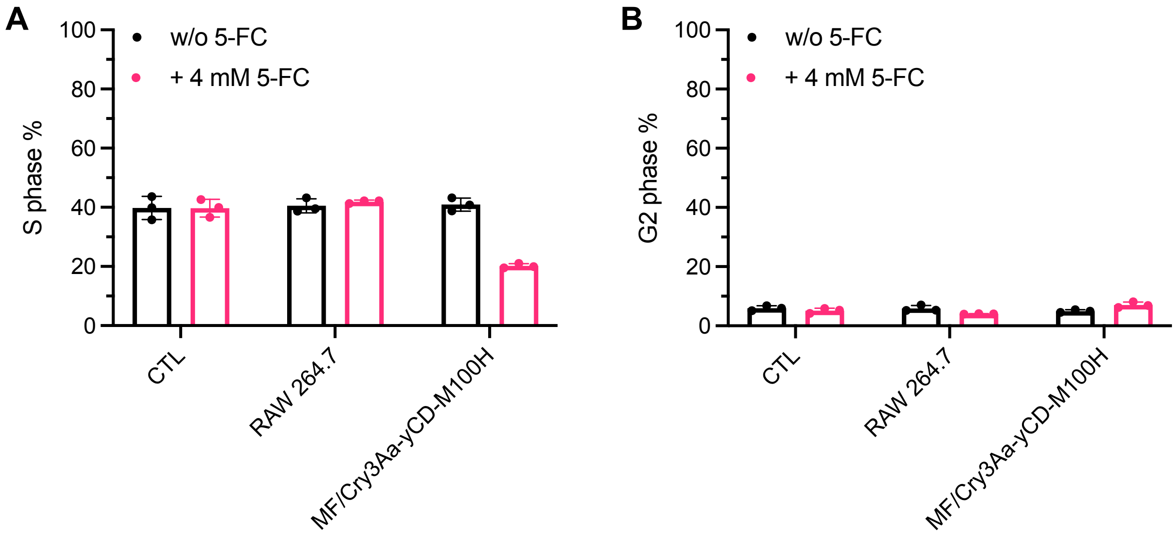

Supplementary Fig. 7. Cell cycle analysis of HT-29 cells after treatment with MF/Cry3Aa-yCD-M100H and 5-FC. The percentage of S phase (A) and G2 phase (B) was analyzed by ModFit (n = 3). Data were shown as mean ± s.d. The result of G1 phase was shown in Fig. 5D.

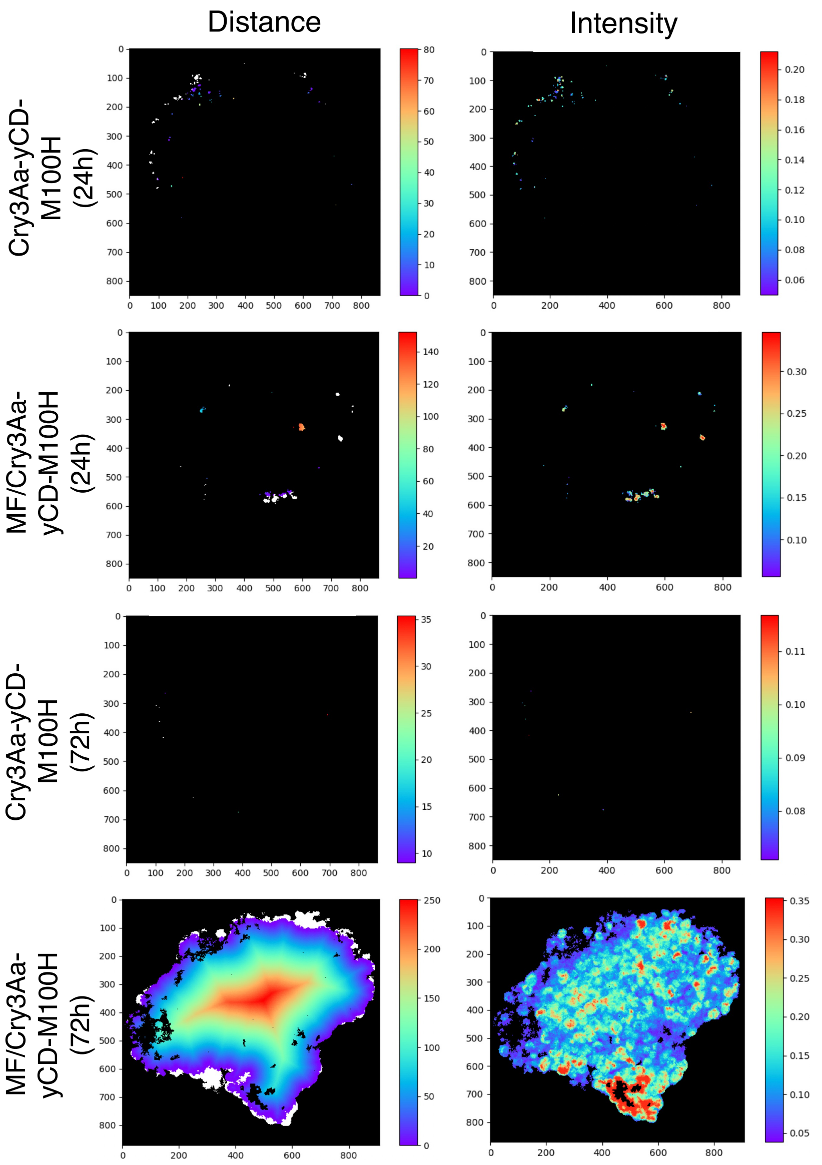

Supplementary Fig. 8. Heat-maps of crystal distance and intensity after treatment of HT-29 spheroids with Alexa-488-labeled Cry3Aa-yCD-M100H or Alexa-488-labeled MF/Cry3Aa-yCD-M100H for 24 or 72 h. Image analysis was performed using CellProfiler.

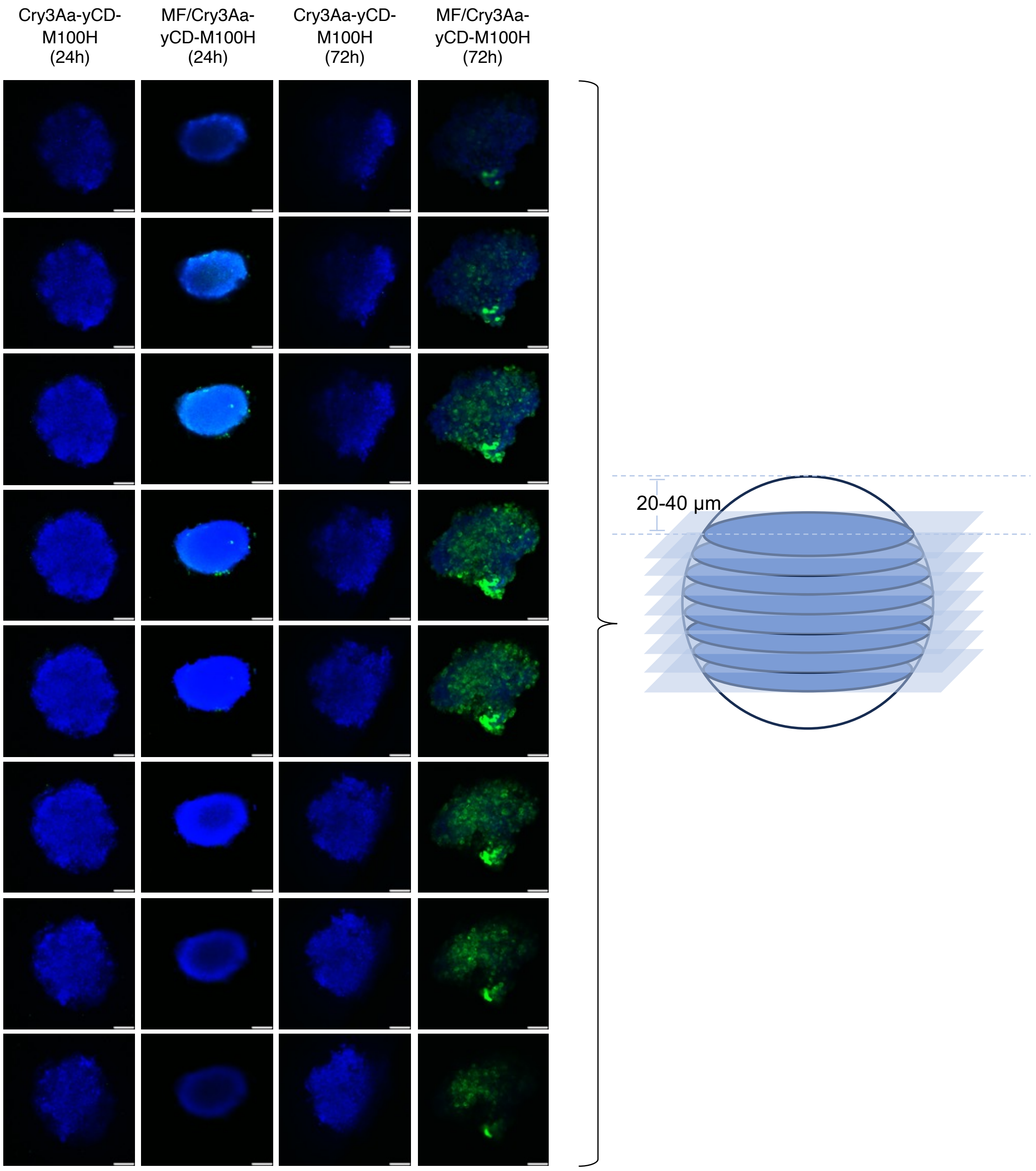

Supplementary Fig. 9. Representative confocal Z-stack images of HT-29 tumor spheroids treated with Alexa-488-labeled Cry3Aa-yCD-M100H or Alexa-488-labeled MF/Cry3Aa-yCD-M100H for 24 or 72 h. A step for Z-stack images is 10 μm; images shown are single slices of the 70-μm thick middle layers from top to bottom view. Scale bar, 75 μm.

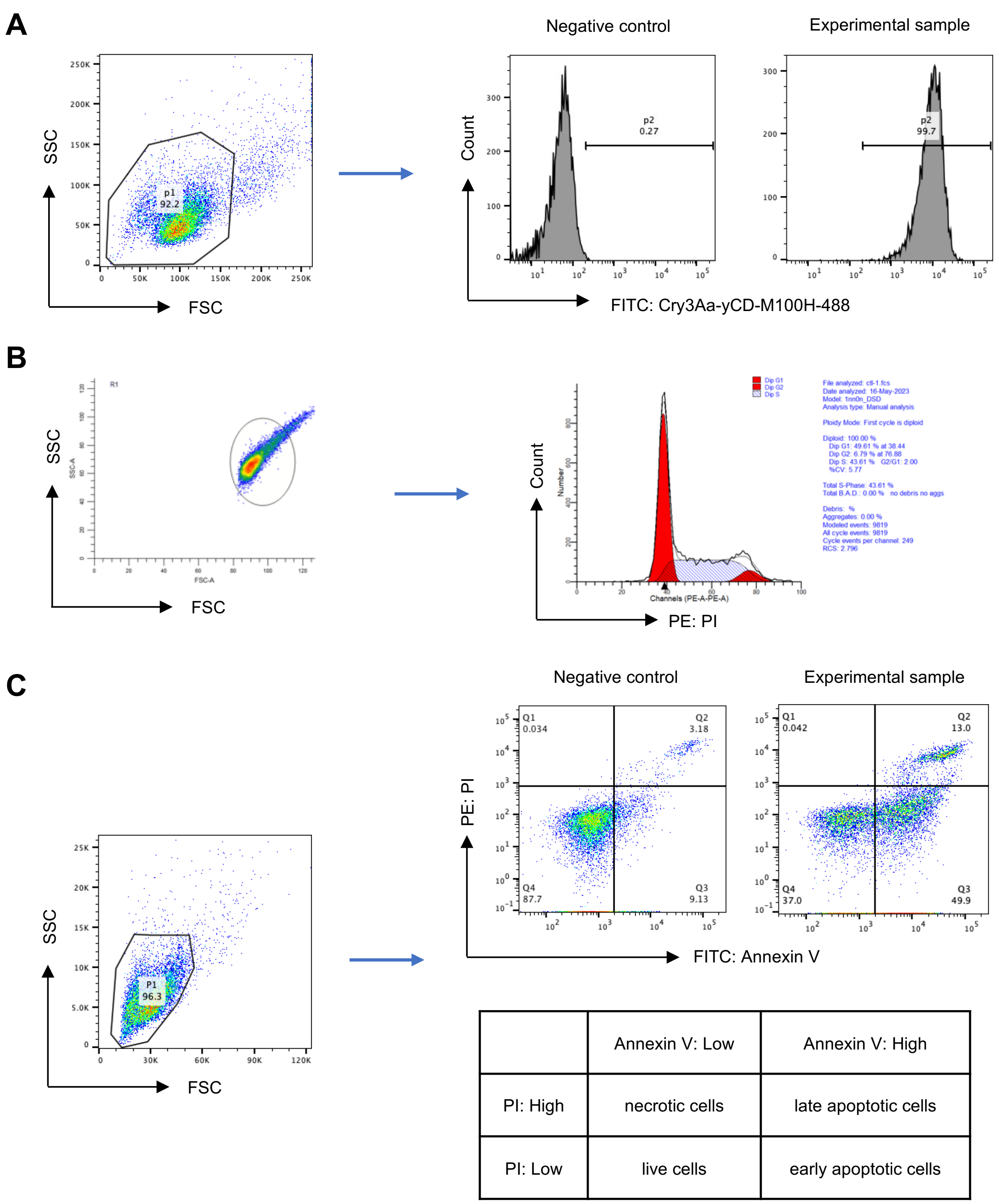

	Annexin V: Low	Annexin V: High
PI: High	necrotic cells	late apoptotic cells
PI: Low	live cells	early apoptotic cells

Supplementary Fig. 10. Gating strategies of cellular uptake assay (A), cell cycle assay (B) and apoptosis assay (C). FSC and SSC signals were used to identify single living cell population.
