# Supplementary material for: An engineered yeast cytosine deaminase with improved catalytic activity and stability for macrophage-mediated enzyme/prodrug therapy
Source: Commun Biol. 2025 Nov 13;8:1562. doi: 10.1038/s42003-025-08931-x (PMC12615592; doi:10.1038/s42003-025-08931-x)
Supplement: Supplementary file 4 — Description of Additional Supplementary Materials [file 42003_2025_8931_MOESM4_ESM.pdf]

Description of Additional Supplementary Files

File name: Supplementary Data 1

Description: The source data behind the main graphs and table 2 in the manuscript.

File name: Supplementary Data 2

Description: The source data used to generate the graphs in the supporting information and the primer sequences designed in this study
