# Supplementary material for: An engineered yeast cytosine deaminase with improved catalytic activity and stability for macrophage-mediated enzyme/prodrug therapy
Source: Commun Biol. 2025 Nov 13;8:1562. doi: 10.1038/s42003-025-08931-x (PMC12615592; doi:10.1038/s42003-025-08931-x)
Supplement: Supplementary file 5 — Reporting summary [file 42003_2025_8931_MOESM5_ESM.pdf]

nature portfolio

Corresponding author(s): Marianne M. Lee; Michael K. Chan

Last updated by author(s): Sep 4, 2025

Reporting Summary

Nature Portfolio wishes to improve the reproducibility of the work that we publish. This form provides structure for consistency and transparency in reporting. For further information on Nature Portfolio policies, see our Editorial Policies and the Editorial Policy Checklist.

Statistics

For all statistical analyses, confirm that the following items are present in the figure legend, table legend, main text, or Methods section.

n/a	Confirmed	
☐	☒	The exact sample size (*n*) for each experimental group/condition, given as a discrete number and unit of measurement
☐	☒	A statement on whether measurements were taken from distinct samples or whether the same sample was measured repeatedly
☐	☒	The statistical test(s) used AND whether they are one- or two-sided<br>*Only common tests should be described solely by name; describe more complex techniques in the Methods section.*
☒	☐	A description of all covariates tested
☐	☒	A description of any assumptions or corrections, such as tests of normality and adjustment for multiple comparisons
☐	☒	A full description of the statistical parameters including central tendency (e.g. means) or other basic estimates (e.g. regression coefficient) AND variation (e.g. standard deviation) or associated estimates of uncertainty (e.g. confidence intervals)
☐	☒	For null hypothesis testing, the test statistic (e.g. F, t, r) with confidence intervals, effect sizes, degrees of freedom and P value noted<br>*Give P values as exact values whenever suitable.*
☒	☐	For Bayesian analysis, information on the choice of priors and Markov chain Monte Carlo settings
☒	☐	For hierarchical and complex designs, identification of the appropriate level for tests and full reporting of outcomes
☒	☐	Estimates of effect sizes (e.g. Cohen's *d*, Pearson's *r*), indicating how they were calculated

Our web collection on statistics for biologists contains articles on many of the points above.

Software and code

Policy information about availability of computer code

Data collection	BD FACSuite (1.0.6)
Data analysis	Fiji (2.14.0), FlowJo (10.4), ModFitLT (5.0.9), GraphPad Prism (10.1.1), CellProfiler (4.2.5), HKL2000, PHASER, Refmac5, Coot, PDB_REDO

For manuscripts utilizing custom algorithms or software that are central to the research but not yet described in published literature, software must be made available to editors and reviewers. We strongly encourage code deposition in a community repository (e.g. GitHub). See the Nature Portfolio guidelines for submitting code & software for further information.

Data

Policy information about availability of data

All manuscripts must include a data availability statement. This statement should provide the following information, where applicable:

- Accession codes, unique identifiers, or web links for publicly available datasets
- A description of any restrictions on data availability
- For clinical datasets or third party data, please ensure that the statement adheres to our policy

The structural information have been deposited in the Protein Data Bank under accession codes 9K1K.

Research involving human participants, their data, or biological material

Policy information about studies with human participants or human data. See also policy information about sex, gender (identity/presentation), and sexual orientation and race, ethnicity and racism.

Reporting on sex and gender	No human participants in this study
Reporting on race, ethnicity, or other socially relevant groupings	No human participants in this study
Population characteristics	No human participants in this study
Recruitment	No human participants in this study
Ethics oversight	No human participants in this study

Note that full information on the approval of the study protocol must also be provided in the manuscript.

Field-specific reporting

Please select the one below that is the best fit for your research. If you are not sure, read the appropriate sections before making your selection.

☒ Life sciences ☐ Behavioural & social sciences ☐ Ecological, evolutionary & environmental sciences

For a reference copy of the document with all sections, see nature.com/documents/nr-reporting-summary-flat.pdf

Life sciences study design

All studies must disclose on these points even when the disclosure is negative.

Sample size	No sample size calculation was performed
Data exclusions	No data were excluded from the analyses
Replication	All attempts on replication were successful
Randomization	No randomization applied
Blinding	No blinding applied

Reporting for specific materials, systems and methods

We require information from authors about some types of materials, experimental systems and methods used in many studies. Here, indicate whether each material, system or method listed is relevant to your study. If you are not sure if a list item applies to your research, read the appropriate section before selecting a response.

Materials & experimental systems

n/a	Involved in the study	
☒	☐	Antibodies
☐	☒	Eukaryotic cell lines
☒	☐	Palaeontology and archaeology
☒	☐	Animals and other organisms
☒	☐	Clinical data
☒	☐	Dual use research of concern
☒	☐	Plants

Methods

n/a	Involved in the study	
☒	☐	ChIP-seq
☐	☒	Flow cytometry
☒	☐	MRI-based neuroimaging

Eukaryotic cell lines

Policy information about cell lines and Sex and Gender in Research

Cell line source(s)	Human colorectal carcinoma HT-29 cells and mouse breast cancer cells 4T1 were gifts from Prof. Thomas Y. C. Leung (The Hong Kong Polytechnic University, HKSAR), and mouse leukemic monocyte macrophage RAW 264.7 cells from Dr. Daniel Lee

	(The Hong Kong Polytechnic University, HKSAR).
Authentication	None of the cell lines used were authenticated.
Mycoplasma contamination	Cell lines were not tested for mycoplasma contamination
Commonly misidentified lines (See ICLAC register)	No commonly misidentified lines

Plants

Seed stocks	No plants used in this study
Novel plant genotypes	No plants used in this study
Authentication	No plants used in this study

Flow Cytometry

Plots

Confirm that:

- [x] The axis labels state the marker and fluorochrome used (e.g. CD4-FITC).
- [x] The axis scales are clearly visible. Include numbers along axes only for bottom left plot of group (a 'group' is an analysis of identical markers).
- [ ] All plots are contour plots with outliers or pseudocolor plots.
- [x] A numerical value for number of cells or percentage (with statistics) is provided.

Methodology

Sample preparation	Details on sample preparation can be found in method section.
Instrument	Cells were analyzed on a BD FACSVerse flow cytometer
Software	Data were analyzed using FlowJo software or ModFitLT
Cell population abundance	The cell abundance in post-sort fraction was around 10,000 cells.
Gating strategy	FSC/SSC was used to determine the live cells .Cells were sorted using fluorescence-activated cell sorting (FACS). Negative sample (without staining) was used as a threshhold to gate positive signals.

- [x] Tick this box to confirm that a figure exemplifying the gating strategy is provided in the Supplementary Information.
